# Quality of vital sign monitoring during obstetric hospitalizations at a regional referral and teaching hospital in Uganda: an opportunity for improvement

**DOI:** 10.11604/pamj.2021.38.252.21749

**Published:** 2021-03-11

**Authors:** Godfrey Rwambuka Mugyenyi, Joseph Ngonzi, Blair Johnson Wylie, Jessica Elizabeth Haberer, Adeline Adwoa Boatin

**Affiliations:** 1 Department of Obstetrics and Gynecology, Mbarara University of Science and Technology, Mbarara, Uganda,; 2 Department of Obstetrics and Gynecology, Beth Israel Deaconess Medical Center, Boston, MA, USA,; 3 Harvard Medical School, Boston, USA,; 4 Center for Global Health, Massachusetts General Hospital, Boston, MA, USA,; 5 Department of Medicine, Harvard Medical School, Boston, MA, USA,; 6 Department of Obstetrics and Gynecology, Massachusetts General Hospital, Harvard Medical School, Boston, MA, USA

**Keywords:** Vital sign monitoring, labor and delivery, postpartum care, childbirth, quality of care

## Abstract

**Introduction:**

vital sign monitoring is a key component of safe facility-based obstetric care. We aimed to assess quality of care around vital sign monitoring during obstetric hospitalizations in a tertiary-care facility in a resource-limited setting.

**Methods:**

retrospective review of obstetric records at a tertiary care facility. We assessed documentation of vital signs including fetal and maternal heart rate, and maternal blood pressure, temperature, oxygen saturation and urine output. The primary outcome was the quality of vital sign monitoring (high- versus low-quality based on frequency of monitoring). We compared quality of monitoring with timing of admission, presence of complication, and delivery mode using chi-squared tests.

**Results:**

among 360 records of obstetric admissions (94% of a planned random sample), 96% documented a delivery. Of these, 8% of pregnant women and 11% of postpartum women had high-quality vital sign monitoring documented on initial evaluation at admission. For women delivering during the hospitalization, 0.8% of women delivering had high-quality monitoring in the first four hours postpartum, with higher rates of high-quality monitoring in women delivering vaginally compared to those delivered by cesarean (1.4% versus 0%, p<0.001). There were no differences in rates of quality monitoring by time of admission, or obstetric complication.

**Conclusion:**

very few obstetric hospitalizations had high-quality vital sign monitoring. Attention towards improving vital sign monitoring is a critical need.

## Introduction

Promoting facility-based childbirth in areas of high maternal mortality is a key strategy to prevent maternal deaths, which continue to be concentrated in sub-Saharan Africa and South-East Asia [1]. However, despite increases in the proportion of women delivering at facilities, reductions in mortality have not been as rapid as expected. Gaps in quality of care at facilities are thought to account for up to 50% of preventable deaths [2-4].

Vital sign (VS) monitoring is a critical component of high quality facility-based obstetric care and often is the first step to identify maternal complication and intervene when needed [5,6]. VS abnormalities are often seen in common obstetric complications, including the most common causes of maternal mortality: hemorrhage, hypertensive disease, and sepsis [7]. For example, hemorrhagic shock can be detected through elevated heart rate (HR), lowered blood pressure (BP), and low urine output (UOP). Similarly, abnormalities in BP and UOP may indicate worsening hypertensive disease. In such clinical scenarios, prompt treatment can avert more dangerous sequelae and ultimately death. Early warning systems, which are increasingly being adopted to improve timeliness in the recognition and management of obstetric complications, rely on VS assessment to score and triage patients [8,9].

Along with competing priorities on busy clinical wards, a shortfall in health care providers may mean that for women undergoing childbirth in facilities or hospitalized for other obstetric indications, VS monitoring may be inadequate. Studies assessing partograph use at facilities in sub-Saharan Africa have demonstrated low completion rates with incomplete fetal and maternal monitoring [10-13]. However, data is lacking on the completeness of vital sign monitoring throughout an obstetric hospitalization, i.e. at other time points than the intrapartum period, and in particular in the immediate postpartum period, when up to 45% of maternal deaths occur [14]. As obstetric complications can occur in both the antepartum, and in the immediate postpartum period (which the partograph does not capture), we aimed to understand the quality of monitoring for the duration of an obstetric admission. In this study, we performed a retrospective review to determine the frequency and quality of vital sign monitoring during obstetric hospitalizations to a tertiary care hospital in Uganda. Our secondary objective was to assess if the hospitalization day (weekend versus weekday), presence of complications or mode of delivery (for parturients) was associated with differences in the proportion of women receiving high quality monitoring.

## Methods

**Setting:** this is a retrospective review of maternal records from 2013 at Mbarara Regional Referral Hospital (MRRH), a publicly-funded teaching hospital in Southwestern Uganda. VS monitoring is performed by medical students, trainee doctors and midwives. VS are recorded in admission and progress notes and partographs. In 2013, the hospital employed 14 obstetricians and 22 midwives and performed ~10,000 deliveries with a maternal mortality rate of 270 per 100,000 livebirths, caesarean rate of 39% and perinatal mortality rate of 56 per 1,000 births.

**Chart selection:** we obtained the medical records numbers of all women admitted to MRRH maternity ward from a surgical services quality assessment registry created to track surgical outcomes at MRRH. All maternity ward hospitalizations were captured using this registry between 2013 and 2014 and coded by admission diagnosis [15,16]. The registry included details on surgical procedures and hospital outcomes but did not include clinical documentation such as vital signs. We therefore used the registry only to identify obstetric hospitalizations. From this registry we selected all obstetric hospitalizations during 2013. Obstetric hospitalizations included women admitted for antepartum (i.e., prelabor), intrapartum and postpartum indication. Using a random sample generator (Excel Version 16.29.1), we selected a random sample (3.5%, n=384) of records.

**Sample size estimation:** we powered our study to provide a reasonable estimate of our primary outcome: the proportion of women with high quality vital sign monitoring at chosen time periods. As prior estimates of this proportion of women with documented high-quality vital sign assessment for our chosen time periods was unknown, we based our sample size calculation assuming an estimated proportion i.e., P of 0.5 which is the recommended proportion when a population proportion is unknown. This approach provides the largest sample size for a given precision and confidence level [17]. Using this proportion, a sample of 384 was thus considered as as sufficient to estimate the proportion level with a precision level of 0.05 (i.e. proportion estimate ± 5%) and 95% confidence level [17].

**Data variables:** from retrieved medical charts we extracted data on date and timing of admission, labor status (early labor: < 4cm cervical dilation; active labor: 4-10cm cervical dilation; second stage of labor; or postpartum) and VS documented including fetal HR if pregnant on admission and maternal HR, BP, respiratory rate (RR), temperature (Temp), UOP and oxygen saturation. UOP was assessed only in the postpartum period. We noted if VS were documented at admission, in the first four hours postpartum, and on each subsequent postpartum day until discharge. Documented complications were also extracted, including hypertensive disorders of pregnancy (HDPs), antepartum hemorrhage, chorioamnionitis, uterine rupture, postpartum haemorrhage (PPH), sepsis, wound infection or dehiscence, re-operation after a caesarean or laparotomy following a vaginal delivery, peripartum hysterectomy, intensive care unit (ICU) admission, and maternal or perinatal (stillbirth or neonatal) death. To assess whether staffing volumes differed by timing of shift and day of week, we also reviewed the number of midwives available per shift including night/day and weekday/weekend from staffing logbooks kept by the head midwife.

**Outcomes:** our primary outcome was quality of VS monitoring, dichotomized as high- and low-quality based on frequency of monitoring. For pregnant women at admission, we defined high-quality monitoring as the assessment of a minimum of fetal HR, maternal HR, BP and Temp (Table 1). All other women with fewer assessments were categorized as having low-quality monitoring. For women who were postpartum at admission, we defined high-quality monitoring as the assessment of maternal HR, BP and Temp, and categorized all other women having fewer assessments as having low-quality monitoring. We also applied this definition in the immediate four hours postpartum and on subsequent postpartum days for women delivering at the hospitals. These definitions are based on professional guidelines from obstetric societies, including guidance from the Uganda Ministry of Health [6,18-20]. Although we did not include UOP in our definitions of high-quality monitoring, we also assessed the extent to which it was documented. We restricted our analysis of VS documentation beyond postpartum depression (PPD) 0 to records where an admission date and discharge date were recorded, as this confirmed inpatient status for those women. We also assessed if clinical documentation (progress note) was present in the postpartum period. Given differences in length of stay by mode of delivery, we examined VS documentation on PPD 1 only for women delivering vaginally and on PPD 1 to 3 for those delivered by caesarean. A sensitivity analysis was performed with the exclusion of Temp as metric for high-quality monitoring at all three time points. Our secondary objective was to assess if quality of vital sign monitored varied by 1) day of week, 2) mode of delivery and 3) whether or not the woman had a complication. For the exposure of day of week, we dichotomized this into weekday vs weekend. Mode of delivery was dichotomized into vaginal delivery vs cesarean delivery and only assessed for women delivering during the hospitalization in the postpartum period (i.e., women with admitted for antepartum indications and postpartum indications were excluded from this analysis). Complication status was dichotomized into women with one or more complications and women without any complications. We measured the mean number of nursing staff by day of shift (i.e., weekday versus weekend) to assess if this differed by day of the week, however we did not directly compare quality of vital sign monitoring to mean number of staff.

**Table 1:** eligibility criteria, time points for vital sign monitoring evaluation and definitions of high -quality vital sign monitoring

Women included in study	Time points vital signs evaluated	Definition of high-quality vital sign monitoring
Antepartum indication for admission	Initial evaluation at admission	Fetal Heart Rate Maternal Heart Rate Maternal Blood Pressure Maternal Temperature
Intrapartum indication for admission	Initial evaluation at admission	Fetal Heart Rate Maternal Heart Rate Maternal Blood Pressure Maternal Temperature
Postpartum indication for admission	Initial evaluation at admission	Maternal Heart Rate Maternal Blood Pressure Maternal Temperature
	Subsequent hospital days	Maternal Heart Rate Maternal Blood Pressure Maternal Temperature
Women delivering during hospitalization	Initial evaluation at admission	Fetal Heart Rate Maternal Heart Rate Maternal Blood Pressure Maternal Temperature
	Immediate postpartum period (first 4 hours after delivery)	Maternal Heart Rate Maternal Blood Pressure Maternal Temperature
	Subsequent hospital/postpartum days	Maternal Heart Rate Maternal Blood Pressure Maternal Temperature

**Statistical analysis**: we calculated the proportion of women with high- and low-quality VS monitoring on admission, in the immediate four hours postpartum and on each postpartum day. Using the chi-squared test, we compared these proportions between women undergoing caesarean versus vaginal delivery, in women with and without complications (one or more), and day of the week (i.e., weekend versus weekend). Mean nursing staff present were summarized by day of shift (i.e. weekend versus weekend). Data was analyzed using Stata version 13 (Statacorp, College Station, TX, USA).

## Results

### Participant characteristics

**Figure 1:**
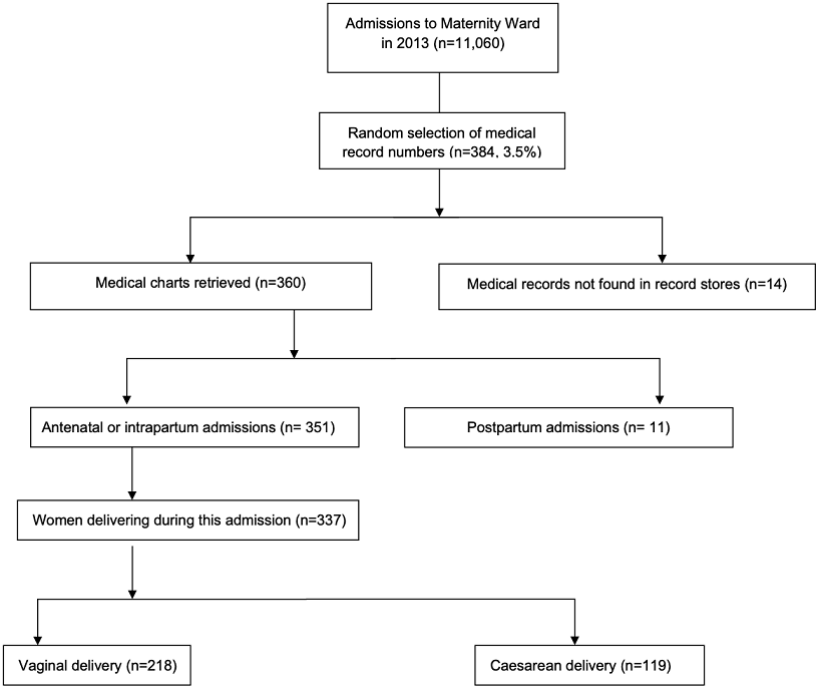
flow diagram of medical record chart selection, retrieval and admission outcomes

A total of 11,060 admissions to the MRRH maternity ward were recorded in 2013 (Figure 1). Of the 384 medical records numbers selected randomly, 360 (93.8%) charts were retrieved; the remaining charts could not be located. Of the 360 charts, 97.5% (n=351) were of women admitted during the antenatal or intrapartum period and 2.5% (n=9) were admitted postpartum. Of the 351 women pregnant at the time of admission, on initial evaluation, 11.6% (n=41) had no evidence of labor, 26.2% (n=92) were in early labor, 41.6% (n=146) in active labor and 20.5% (n=72) in the second stage of labor. A total of 337 (96.1%) records reported an intrapartum period with a delivery occurring. Of these, 119 (35.3%) women had a caesarean delivery, 5 (1.5%) had a vacuum delivery. The remainder were spontaneous vaginal deliveries.

### Vital sign assessment

The proportion of charts with documented VS on admission are presented in Table 2. Eight percent (n=29/351) of pregnant women and 11% of postpartum women (n=1/9) had high-quality monitoring on admission. The most common individually checked VS was fetal HR, documented in 83% (n=291/351) of charts. Maternal HR, BP and Temp were documented in 48% (n=170/351), 50% (n=177/351), and 11% (n=39/315) of charts, respectively. Among postpartum admissions, the most frequently documented VS was maternal HR in 67% (n=6/9) of admissions, followed by BP, which was documented in 44% (n=4/9).

**Table 2:** vital sign monitoring on admission

Vital Sign	Pregnant on admission N (%) N=351	Postpartum on admission N (%) (N=9)
High quality Monitoring a	29 (8.3)	1 (11.1)
Heart Rate	170 (48.4)	6 (66.7)
Blood Pressure	177 (49.6)	4 (44.4)
Temperature	39 (11.1)	1 (11.1)
RR	21 (6.0)	0
Oxygen Saturation	0	0
Fetal Heart Rate	291 (82.9)	-

a Includes maternal heart rate, blood pressure, temperature and fetal heart rate in pregnant women

For women delivering in the hospital, very few charts had documentation of VS in the first four hours postpartum (Table 3). Less than 1% (n=3) of the 337 women delivering had documentation of high-quality monitoring in the first four hours. More women who delivered vaginally had high-quality monitoring in the first four hours after delivery compared to those delivered by caesarean at 1.4 % vs 0% (p<0.001), but this was still a rare event. In a sensitivity analysis with Temp excluded, the proportion with high-quality VS monitoring increased to 5% (n=16) of women. The most frequent VS measured in the first four hours post-delivery was BP, documented in 7% of women, followed by HR, documented in 4% of women. Temp and RR were documented in 0.9% (n=3) of women. No woman had UOP documented at any time point in the postpartum period.

**Table 3:** vital sign monitoring in the postpartum period (n/%) over time and by mode of delivery

Vital Sign	Cesarean (N=119)				Vaginal (N=218)	
	First 4 hours post op (N=119)	Postpartum day 1 (N=86)	Postpartum day 2 (N=84)	Postpartum day 3 (N=69)	First 4 hours post op (N=219)	Postpartum day 1 (N=15)
High quality Monitoring a	0	4 (3.6)	3 (2.8)	3 (3.4)	3 (1.4)	1 (0.8)
HR + BP	2(1.7)	32 (29.1)	19 (17.8)	9 (10.1)	14 (6.4)	5 (4.0)
Heart Rate	2 (1.7)	40 (36.4)	28 (26.2)	19 (21.4)	14 (6.6)	6 (4.8)
Blood Pressure	4 (4.2)	38 (34.6)	26 (3)	10 (11.2)	19 (8.7)	7 (5.7)
Temperature	0 (0)	7 (6.4)	6 (5.6)	3 (3.4)	3 (1.4)	1 (0.8)
RR	1(0.9)	2 (1.8)	1 (0.9)	0	2 (0.9)	2(1.6)
Oxygen Saturation	1(0.8)	1 (0.9)	0	0	1 (0.5)	0
Urine output	0	0	0	0	0 (0)	0

a Includes maternal heart rate, blood pressure and temperature

Data on VS monitoring beyond PPD 0 were available for 86% (n=296) of records. Just over 12% (n=15) of women who delivered vaginally had any clinical documentation on PPD 1; <1% (n=1) of these women had high-quality VS monitoring documented. HR was documented in 4% (n=5), BP 6% (n=7), and Temp 0.8% (n=1). Women delivered by caesarean had clinical documentation in 78% of records on PPD 1, 2, and 3. High-quality monitoring was performed in 3.6% (n=4), 2.8% (n=3) and 3.4% (n=3) of women on PPDs 1, 2, and 3, respectively. Excluding Temp, the proportion with high-quality monitoring rose to 29%, 17.8%, and 10.1% on PPDs 1, 2, and 3, respectively. No UOP was documented in any of the available clinical records.

### Staffing and vital sign documentation

Table 4 summarizes mean number of midwives during different shifts (day, evening, and night). Significantly more midwives were available on weekday compared to weekend day shifts (6.4 versus 4.3, p<0.001) and day shifts compared to evening and night shifts (5.8 versus 2.9 and 3.0, respectively). However, we found no significant difference in the proportion of records with high-quality monitoring for admissions on a weekday versus weekend (7% versus 9%, p=0.5) nor during the first fours after delivery for deliveries on a weekday versus weekend 9% versus 4%, p=0.1).

**Table 4:** nursing staffing in the obstetrics and gynecology department during study period

	Overall mean ±SD	Weekday	Weekend	
Day Shift	5.8 ± 1.6	6.4±1.4	4.3±1.0	P<0.01
Evening Shift	2.9 ± 0.6	3.0±0.5	2.7±0.6	P<0.01
Night shift	3.0 ± 0.4	2.9±0.4	3.0±0.5	P=0.05
Mean number of nurses	11.7 ± 1.8	12.3±1.5	10.0±1.4	P<0.01

### Recorded complications

PPH was the most common complication, occurring in 5% (n=8) of women. HDPs occurred in 6 (1.7%) of women. There was one (0.28%) maternal death, 11 (3.0%) stillbirths and one (0.3 %) neonatal death. One or more complication was recorded in 42 (11.0%) women (Table 5). High-quality monitoring was performed more frequently in women without a complication compared to women with a complication at admission (4.8% versus 8.5%, p=0.4) and in the first fours after delivery (1% vs 0%, p=0.6), however these differences were not statistically significant.

**Table 5:** obstetric complications documented

Complications	N % (n=360)
Hypertensive Disorders of Pregnancy	4 (11.1)
Eclampsia	2 (0.6)
Antepartum hemorrhage	6 (1.7)
Chorioamnionitis	1 (0.3)
Uterine rupture	0
Postpartum hemorrhage	18 (5.0)
Sepsis	1 (0.3)
Wound dehiscence	2 (.6)
Re-operation	0
ICU admission	0
Maternal death	1 (0.3)
Fresh Stillbirth	7 (1.9)
Macerated stillbirth	4 (1.1)
Neonatal death	1 (0.3)
Any Complication (one or more complication)	42 (11%)

## Discussion

In this review, we examined VS documentation for obstetric care in a busy tertiary care facility in Uganda. We found VS documentation rarely met international normative quality standards with <9% of women having high-quality monitoring on admission and <1% of women undergoing delivery having high-quality monitoring in the immediate postpartum period. Notably, among women undergoing caesarean, there was no documentation of any VS in the first four hours after delivery. Mean nurse staffing availability, day of week and presence of an obstetric complication did not significantly influence rates of VS documentation.

Our study findings mirror those seen in other low-resourced obstetric settings. In a trial conducted in India where checklists were tested as an intervention to improve quality of care during facility-based childbirth, maternal BP and Temp were checked in <38% of women in the intervention arm and less than 3% of women in the control arm [21]. However, our findings are somewhat different from a study in the same facility (MRRH) assessing frequency of VS monitoring among non-obstetric patients with severe sepsis in a medical ward. In that study, audits demonstrated that 96% of patients had at least one BP measurement in the first 24 hours of admission, with a steep decline in monitoring over the course of their inpatient stay [22]. This higher level of monitoring observed on admission compared to our findings may be due to differences in staff: patient volumes in different wards. For example, although nursing staffing on the medicine and obstetrics wards is about the same at MRRH, the obstetrics unit often has five-fold higher patient census with a higher turnover.

We found no documentation of VS monitoring in the first four hours after delivery among the 119 women undergoing caesarean in this study. This is noteworthy. In two recent studies, the risk of mortality after caesarean was demonstrated to be 50 times higher in 22 sub-Saharan countries and other low- and middle-income countries compared to high-income countries [23,24]. Although the root causes of higher mortality are not yet understood, these findings point to the importance of perioperative monitoring for women undergoing caesarean and our study demonstrates a dire lack of such monitoring. In high-resource settings, routine, high frequency VS monitoring around childbirth is considered standard of care. Nursing guidelines in the United States recommend the assessment of maternal HR and BP every 15 minutes for two hours in the immediate postpartum period and more frequently if complications are encountered [6]. To achieve this goal, 1: 1 nursing to patient ratio is recommended during that time, which is similar to the level of care found in intensive care units. Our findings in this resource-limited setting indicate that women receive far less monitoring, even when complications are present. Given the staffing availability, this situation is unsurprising. On the most robustly staffed weekday shift, we found no more than seven nurses, with responsibilities for covering all antepartum, intrapartum, and postpartum patients, translating to a nurse-to-patient ratio of approximately to 1: 25 at best. Furthermore, monitoring after caesarean may be hard to implement in the absence of a postoperative anesthesia care unit combined with delays in transport to the postpartum ward.

Addressing this gap in care requires innovative solutions, as we are unlikely to see large increases in nursing staff in the near future. Current projections are that shortages in health providers are likely to worsen, rising from a current shortfall of 7.3 million to 18 million by 2030 and with deficits concentrated in low- and middle- income countries [25]. As such, new methods of monitoring are needed to achieve the levels of high-quality monitoring considered normative standards. Consideration of non-traditional staff, use of family members and advances in wearable technology may be strategies to improve monitoring during and around childbirth [26].

Our study has several limitations. Firstly, our assessment of VS monitoring and clinical outcomes is limited to what is documented in the medical record; therefore, it is possible that some VS monitoring occurred without documentation or that the medical records selected were missing components of the chart. We obtained a random selection of charts to prevent any systematic bias in this regard. However, we noted that over 50% of charts (primarily those of women delivering vaginally) had no documentation of any care received on the first postpartum day one. This finding could imply either poor or lost documentation or represent true gaps in clinical care. However, this data is still relevant to quality of care; deficits in the documentation of VS at one point during admission may impact care at a later point, as clinicians may need to understand the trends in VS to make clinical inferences. Secondly, our sample size was based on an estimate frequency of 50% VS assessment. Given the much lower rates found in this review, a large sample size would be needed to detect clinically significant differences in monitoring by mode of delivery, day of assessment and complication. Lastly, we were unable to compare staff to patient ratios which may be more informative of the workload. This information should be assessed in future prospective work assessing VS monitoring.

## Conclusion

In conclusion, most obstetric admissions in this busy tertiary care facility had low-quality VS monitoring, if any monitoring at all. As a foundation of inpatient clinical care, particularly at a referral center, this finding provides a clear target and opportunity for improvement in quality of care. Current global strategies to improve maternal health continue to favor facility birth, with attention still focused on encouraging women to access facilities for delivery. Concomitant attention to improving the quality of care delivered at such facilities is therefore also essential with emphasis placed on how standards can be improved despite resource limitations.

### What is known about this topic

- Vital sign monitoring is a key component on safe facility-based childbirth;
- In low-resource settings deficits in vital sign monitoring in non-obstetric populations have been described.

### What this study adds

- Evidence demonstrating a critical deficit in postpartum care for women undergoing facility-based delivery;
- Identifies a concrete area for improved quality of care during facility-based childbirth.
